# Structural Basis for Plant MADS Transcription Factor Oligomerization

**DOI:** 10.1016/j.csbj.2019.06.014

**Published:** 2019-06-14

**Authors:** Xuelei Lai, Hussein Daher, Antonin Galien, Veronique Hugouvieux, Chloe Zubieta

**Affiliations:** Laboratoire de Physiologie Cellulaire et Végétale, CNRS, Univ. Grenoble Alpes, CEA, INRA, IRIG, Grenoble, France

**Keywords:** MADS transcription factors, Oligomerization, Floral development, Arabidopsis

## Abstract

MADS transcription factors (TFs) are DNA binding proteins found in almost all eukaryotes that play essential roles in diverse biological processes. While present in animals and fungi as a small TF family, the family has dramatically expanded in plants over the course of evolution, with the model flowering plant, *Arabidopsis thaliana*, possessing over 100 type I and type II MADS TFs. All MADS TFs contain a core and highly conserved DNA binding domain called the MADS or M domain. Plant MADS TFs have diversified this domain with plant-specific auxiliary domains. Plant type I MADS TFs have a highly diverse and largely unstructured Carboxy-terminal (C domain), whereas type II MADS have added oligomerization domains, called Intervening (I domain) and Keratin-like (K domain), in addition to the C domain. In this mini review, we describe the overall structure of the type II “MIKC” type MADS TFs in plants, with a focus on the K domain, a critical oligomerization module. We summarize the determining factors for oligomerization and provide mechanistic insights on how secondary structural elements are required for oligomerization capability and specificity. Using MADS TFs that are involved in flower organ specification as an example, we provide case studies and homology modeling of MADS TFs complex formation. Finally, we highlight outstanding questions in the field.

## 1 Introduction

MADS transcription factors (TFs) are found in almost all eukaryotes, playing important roles from pheromone sensing in yeast, to muscle development and cell proliferation and differentiation in animals [1] and to floral development in plants [2]. The core MADS DNA-binding domain (DBD) likely evolved from a domain of topoisomerase IIA, an enzyme involved in DNA maintenance [3]. MADS TFs are characterized by this canonical and evolutionarily conserved DBD. The MADS family draws its name from the members, MAINTENANCE OF MINICHROMOSOME1 (MCM, *S. cerivisae*), AGAMOUS (AG, *A. thaliana*), DEFICIENS (DEF *A. majus*) and Serum Response Factor (SRF, *H. sapiens*) [2,[4], [5], [6], [7], [8], [9], [10]]. While present in animals and fungi as a small TF family, plants have dramatically expanded the MADS family from a few MADS TFs in basal phyla such as charophytes (green algae) to over a hundred different members in Arabidopsis [3,11]. In higher plants, MADS TFs act as key regulators in diverse developmental processes including seed germination [[12], [13], [14], [15]], vegetative growth [16,17], transition to flowering [18,19], flower development [9,20,21] and senescence [22,23]. How plant MADS TFs achieve this functional diversity is a fundamental question we are now starting to answer [[24], [25], [26]].

**Fig. 1:**
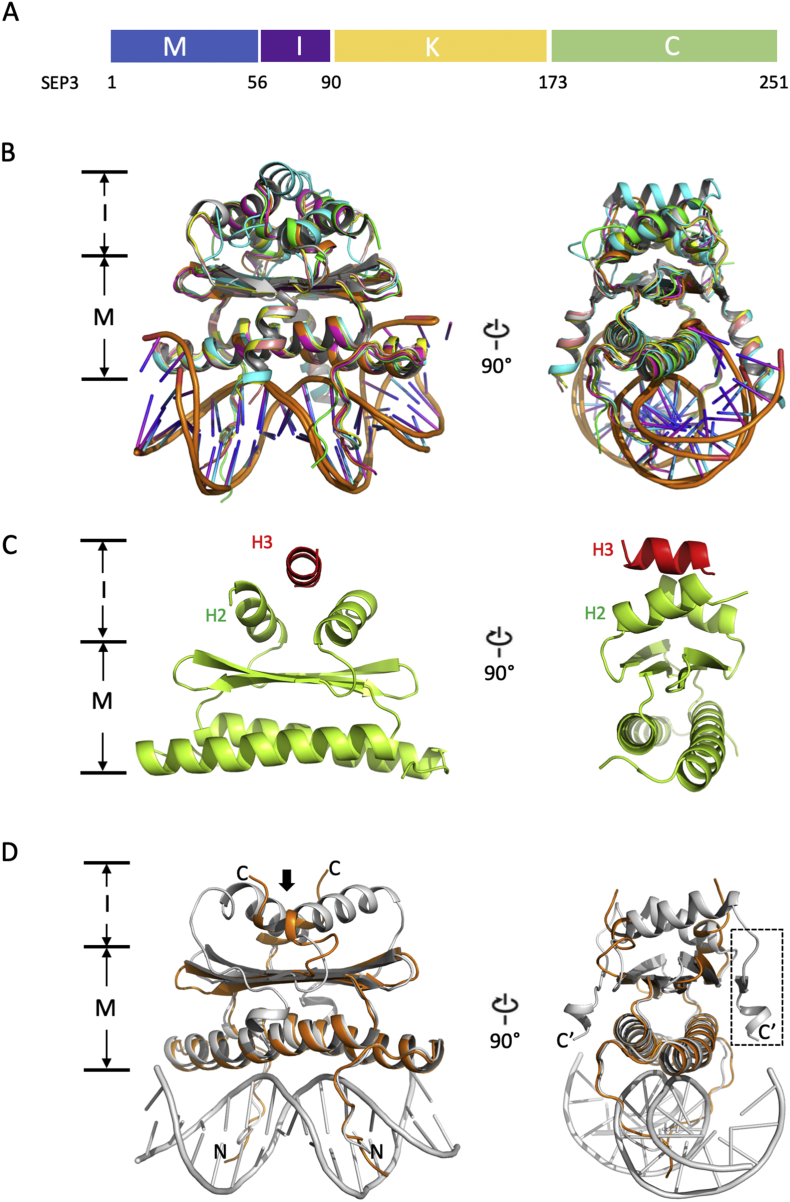
3D model superposition of M domains and I domain extensions of MADS family proteins from yeast and human with plant MI domains modeled. (A) Schematic representation of domain compositions of MIKC type MADS TF using SEP3 amino acid numbering as an example. (B) The coordinates were taken from yeast MCM1 (green, PDB 1MNM; residues 18–91) [47], human SRF (magenta, PDB 1SRS, residues 132–223) [46], human myocyte enhancer factor 2A (MEF2A) (yellow, PDB 3KOV, residues 2–95) [45], human MEF2B (cyan, PDB 1N6J, residues 2–58) [54], human MEF2 chimera (pink, PDB 6BYY, residues MEF2A (1–64 and 91–95) and MEF2B (65–90)) [55]. Two SEP3 models (SEP3-M1 in orange (residues 1–72) and SEP3-M2 in gray (residues 2–89)) were generated from SWISS-MODEL [56], using PDB 1SRS and 6BYY as templates, respectively. (C) Crystal structure of human MEF2B (PDB 6C9L) showing the presence of α-helix, H3 (highlighted in red), on top of H2 helices; Note that H3 in this conformation is only present in one dimer (formed by chain E and F) of the three dimers in the asymmetric unit. (D) Superposition of SEP3 M + I domain models showing different conformation possibilities of the I domain (indicated by a bold arrow). M, M domain; I, I domain; N, N-terminus; C, C-terminus of SEP3-M1; C′, C-terminus of SEP3-M2 (part of I domain is indicated in dash box).

All MADS TFs bind DNA as obligate dimers, recognizing a “CArG” box motif (CC[A/T]_6_GG) [27]. Allowing a single mismatch, this motif is represented over 340,000 times in the Arabidopsis genome and it has been hypothesized that MADS TFs could potentially control up to 30% of genes in Arabidopsis [28]. The complex gene regulation directed by the MADS TFs in plants requires highly specific targeting of the TF to different target genes. In order to achieve this, the canonical MADS DNA binding domain (DBD) is elaborated with additional domains. The plant MADS TF family can be divided into two distinct types, type I and type II [29]. Type I MADS TFs possess the core MADS domain (M domain) and an extended and highly variable Carboxy-terminal domain. Apart from the M domain, type I plant MADS TFs do not have additional conserved and predicted folded domains and will not be further discussed in this review. In contrast, the type II MADS TFs are characterized by a conserved four-domain structure, termed “MIKC” that includes the M domain, the I domain (Intervening domain, a short helical domain involved in dimerization specificity), the K domain (Keratin-like domain, a predicted coiled-coil important for tetramerization), and the C domain (Carboxy-terminal domain, which is a sequence variable and largely unstructured domain involved in transactivation and/or recruitment of other factors) (Fig. 1A). The MIKC MADS are further divided into MIKC^C^ and MIKC^⁎^ based on phylogeny and sequence conservation but retain this modular domain structure [30]. The I and the K domains were acquired during evolution and functionally diversify the conserved MADS DBD by facilitating oligomerisation with other MADS partners [31]. Adding these domains resulted in the development of a large protein–protein interaction (PPI) network consisting of homo- and hetero- dimers and tetramers. The tetrameric MADS complexes formed primarily by MIKC^C^, are plant specific and can bind to DNA cooperatively at two sites [32,33], unlike other eukaryotic MADS TFs that only bind as dimers to a single CArG box [34]. For example, in Arabidopsis, the SEPALLATA (SEP) clade functions as a hub within the MADS PPI network and drives the formation of distinct tetrameric complexes [35,36]. Each complex is sufficient to determine a specific floral organ fate [2,6,20,37]. Extensive studies of the MADS PPI network using yeast “N”-hybrid experiments have revealed complex interaction patterns at both the level of the dimer as well as the tetramer [38,39].

In this review, we describe the overall architecture and structure of Type II MIKC MADS TFs, with a focus on the oligomerization domain based on the recent structural characterization of the SEP3 K domain and extensive mutagenesis studies [[40], [41], [42]]. We further address the structural determinants of K domain oligomerization, key structural motifs and K domain evolution.

## 2 Structure of MIKC MADS TFs

### 2.1 The Highly Conserved MADS Domain

The MIKC MADS TFs are modular multi domain proteins with highly conserved secondary structures (Fig. 1A). The DBD, or M domain, consists of ~55–60 residues and is highly conserved from animals to plants (with sequence identity as high as 40% between *H. sapiens* and *A. thaliana*). In addition to binding DNA, most plant M domains contain a predicted bipartite nuclear localization sequence (NLS) with the consensus sequence (K/R)(K/R)X_10_(K/R)_3–5_ [43,44]. The M domain has been structurally characterized in human and yeast and reveals a conserved fold and highly conserved DNA-binding residues (Fig. 1B) [[45], [46], [47]]. Domain swap experiments in Arabidopsis underscore how highly conserved the M domain is over the course of evolution. These experiments have shown that *APETALA1 (AP1)*, *APETALA3 (AP3)*, *PISTILLATA (PI)* and *AGAMOUS (AG)* chimeras possessing the M domain of *SRF* (yeast) or *MEF2* (human) are able to complement the appropriate mutant phenotype as well as the native gene, and can reproduce the phenotypes exhibited by plants expressing *35S::AP1*, *35S::AP3*, *35S::PI* and *35S::AG*, respectively [48]. These data suggest that either 1) the M domain is dispensable for DNA-binding specificity and ternary factors are required for *in vivo* binding specificity, 2) other MADS TF partners in the plant MADS heteromeric complexes may compensate for the switch in M domain of a single partner and/or 3) the oligomerization domains (*i.e.*, I and K domains) strongly contribute to DNA-binding specificity, perhaps *via* allosteric interactions. It is likely that a combination of these factors plays a part in determining DNA-binding specificity and function. For example, *in vivo* specificity studies using ChIP-seq demonstrated enrichment of non-CArG box motifs for MADS TF binding sites, suggesting a role for ternary factors [49]. Domain swap experiments described below for the I domain have been shown to alter DNA-binding specificity. *In vitro* and *in vivo* studies of the K domain have also demonstrated a role in DNA-binding specificity and *in vivo* function for the plant MIKC MADS [50].

### 2.2 The Flexible and Variable Intervening Domain

The I domain, a small (~25–30 amino acid) linker domain between M and K domains (Fig. 1A), is involved in dimerization specificity based on *in vitro* domain swaps, band shift assays, computational models and yeast 2-hybrid studies [51,52]. This domain exhibits greater sequence diversity than the M or K domains within the plant MIKC family. Based on secondary structure predictions, the I domain is largely α-helical. While the I domain does not exhibit high sequence homology to mammalian MADS, this domain may be structurally homologous its mammalian counterpart.

In the mammalian MADS box containing proteins, such as MEF2B, adjacent to the M domain is a small domain, consisting of two α-helices, called H2 and H3, that helps to stabilize the M domain by sandwiching the β sheet adjacent to the DNA-binding α-helix (Fig. 1C). The presence of flexible loops between H2 and H3 α-helices allows different conformations for the C-terminal α-helix, H3, as has been shown by a recent crystal structure in which one C-terminal MEF2 H3 is flipped up to stack on top of the H2 helices, a novel conformation not see in previous structural studies (Fig. 1C) [53]. This conformation, in which the H3 helix occupies the same protein-binding region as cofactors such as Cabin1, is observed for only one dimer out of three in the asymmetric unit. In addition, the second H3 helix from the partner monomer is completely missing from the structure due to either disorder or possibly *in situ* proteolysis. While the H2 helices form a protein-protein interface for a single helix to bind, this interface is not sufficient to bind two H3 helices and is unlikely to mimic plant MIKC dimers which would present two helices. The different conformations for MEF2 are likely the result of truncated constructs and crystal packing due to inherent flexibility between H2 and H3 and may have limited physiological relevance.

Based on human and yeast structures of MADS TFs, different homology models for plant MI domains can be generated. Using the 3D structure of human MADS TFs (PDB 1SRS and 6BYY), we generated two M domain (with partial I domain) models for SEP3, called SEP3-M1 and SEP3-M2, respectively (Fig. 1D). In both cases, the H2 helix of the I domain is present but with different conformations (indicated by bold arrow in Fig. 1D); for the SEP3-M2 model, a second helix (H3) with a flexible linker is modeled (in dashed box, Fig. 1D). Depending on the conformation and extension direction of the I domain, two distinct structural modes can be modeled that could potentially affect DNA binding through direct DNA contacts *via* the C-terminal extensions of the I domain [40]. Whether the I domain of plant MIKC TFs adopts one of these MEF2 conformations remains to be determined as no MI domain structures from plants are currently available.

### 2.3 The K Domain, a Key Oligomerization Module

MIKC type MADS genes have been reported even in the charophytes, extant green algae that are the closest relatives to the land plants [57]. This suggests that the MIKC type MADS genes evolved by the addition of a K domain to an ancestral MADS gene present in the most recent common ancestor of charophytes and chlorophytes which contained a type II MEF2 MADS gene with an M domain but lacked the IKC domains [29,31,58]. In seed plants, it has been described that the K domain sequences of MIKC type MADS TFs are highly conserved even among sequences sampled from high evolutionary distance [42], suggesting that the overall structure of the K domain is retained among MIKC type MADS TFs over the course of evolution. Thus, the K domain is a highly conserved and defining feature of the MIKC plant MADS TFs.

Based on secondary structure predictions, the K domain was divided into three α-helices, K1, K2 and K3. Extensive mutagenesis studies determined that hydrophobic residues in the K domain were of particular importance for mediating protein-protein interactions and critical for dimerization and tetramerization of MADS TFs involved in floral organ identity. Leucine-zipper motifs in the K1 (position 87–108, PI numbering) and K2 (position 121–135, PI numbering) helices, for example, were shown to be important for AP3/PI heterodimerization, based on extensive mutagenesis studies and yeast 2-hybrid assays [59,60]. Further mutagenesis studies and yeast 3-hybrid assays incorporating the additional MADS partner, SEP3, helped identify residues in the K2 and K3 (position 142–167, PI) helices contributing to tetramerization [59]. These studies provided an important basis for predicting dimer and tetramer interactions. The subsequent 3D crystal structure of the K domain from SEP3 demonstrates that the residues identified through previous mutagenesis studies are either directly contacting the dimer/tetramer interface or are required for maintaining the structural integrity of the K domain helices [40].

**Fig. 2:**
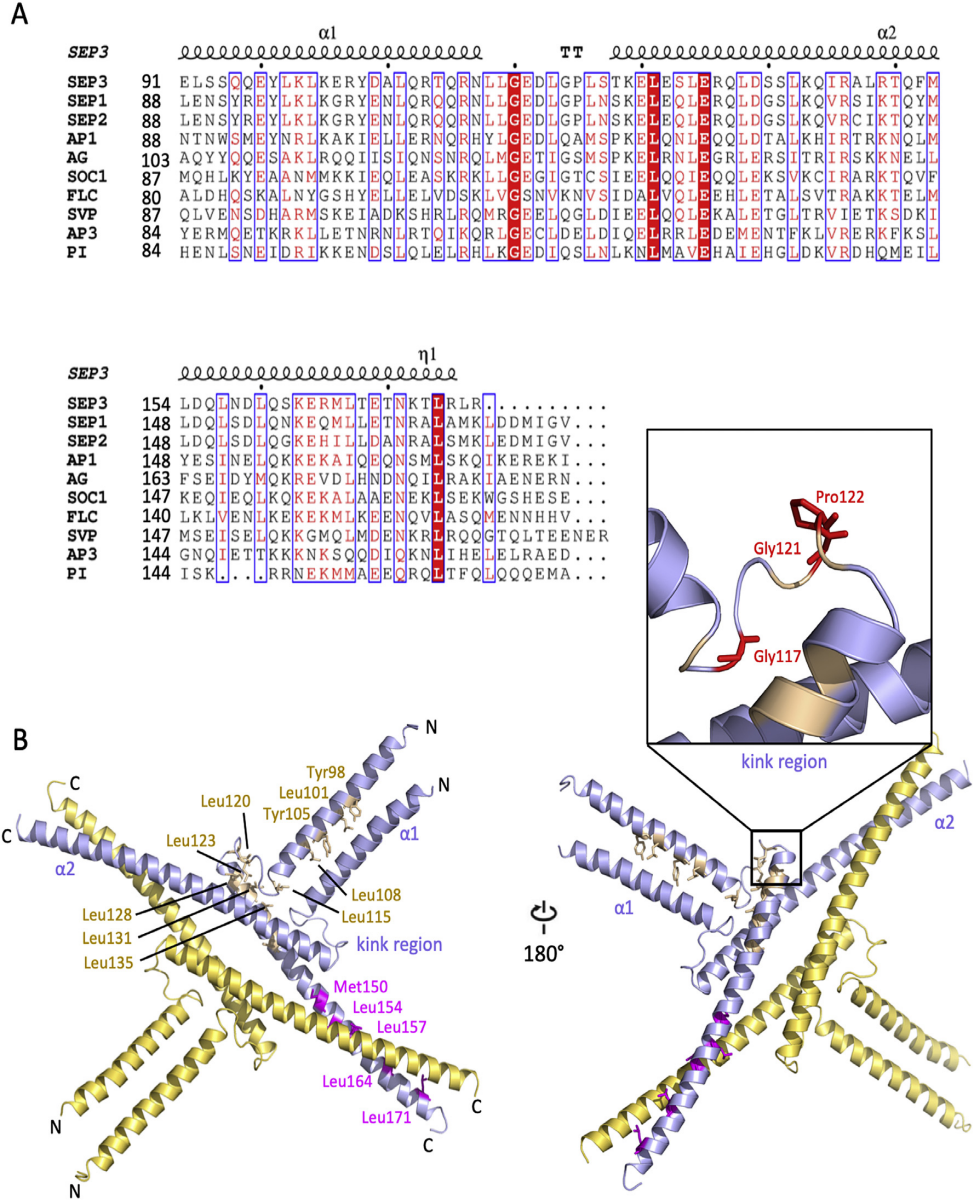
K domain sequence alignment of some representative MADS TFs from Arabidopsis and SEP3 K domain structure. (A) K domain sequence alignment of SEP3, SEP1, SEP2, AP1, AG, SUPPRESSOR OF OVEREXPRESSION OF CONSTANS 1 (SOC1), FLOWERING LOCUS C (FLC), SHORT VEGETATIVE PHASE (SVP), AP3 and PI, using ESPript 3 [65]. TT indicates the kink region that connects α1 and α2. (B) Crystal structure of SEP3 K domain (PDB 4OX0). The residues involved in dimer and tetramer formation are indicated and colored by wheat and magenta, respectively. The insert shows key residues in the kink region colored red.

The crystal structure of the K domain from SEP3 was the first and only structurally characterized oligomerization module of a plant MADS TFs available to date (Fig. 2B) [40]. The crystal structure (residues 75–178) contains the complete K domain (residues 91–173), a small portion of the I domain (residues 75–90) and several residues of the C domain (residues 174–178). Each monomer folds into two amphipathic α-helices, α1 (residues 75–114) and α2 (residues 115–178) separated by a glycine and/or proline rich spacer (referred to as the “kink region”) of approximately 5–10 amino acids (Fig. 2B). Two monomers interact *via* their N-terminal α-helices to form a dimer and two dimers form a tetramer *via* their C-terminal α-helices. Thus, the biological assembly of SEP3 in the crystal structure is a homotetramer or a dimer of dimers. Sequence alignments of representative type II MADS TFs from Arabidopsis reveal that the amino acids in the kink region are variable but contain glycines and/or proline residues, resulting in a “break” between the α1 and α2 helices (Fig. 2A). This break is characterized by either added flexibility due to the presence of glycine residues, which are able to sample more conformational space, or proline residues which break the α–helical secondary structure and help force the two helices apart in a more rigid conformation. Thus, the α1 and α2 helices of the K domain are able to form two distinct coiled coils, allowing dimerization and tetramerization. The coiled coil motif is a well-established secondary structure element that can be found in many different protein families and is often involved in protein–protein interactions and/or DNA-binding [[61], [62], [63], [64]]. Plants have thus co-opted a versatile domain to expand their repertoire of protein-protein interactions.

#### 2.3.1 The K Domain Is a Leucine Zipper

**Fig. 3:**
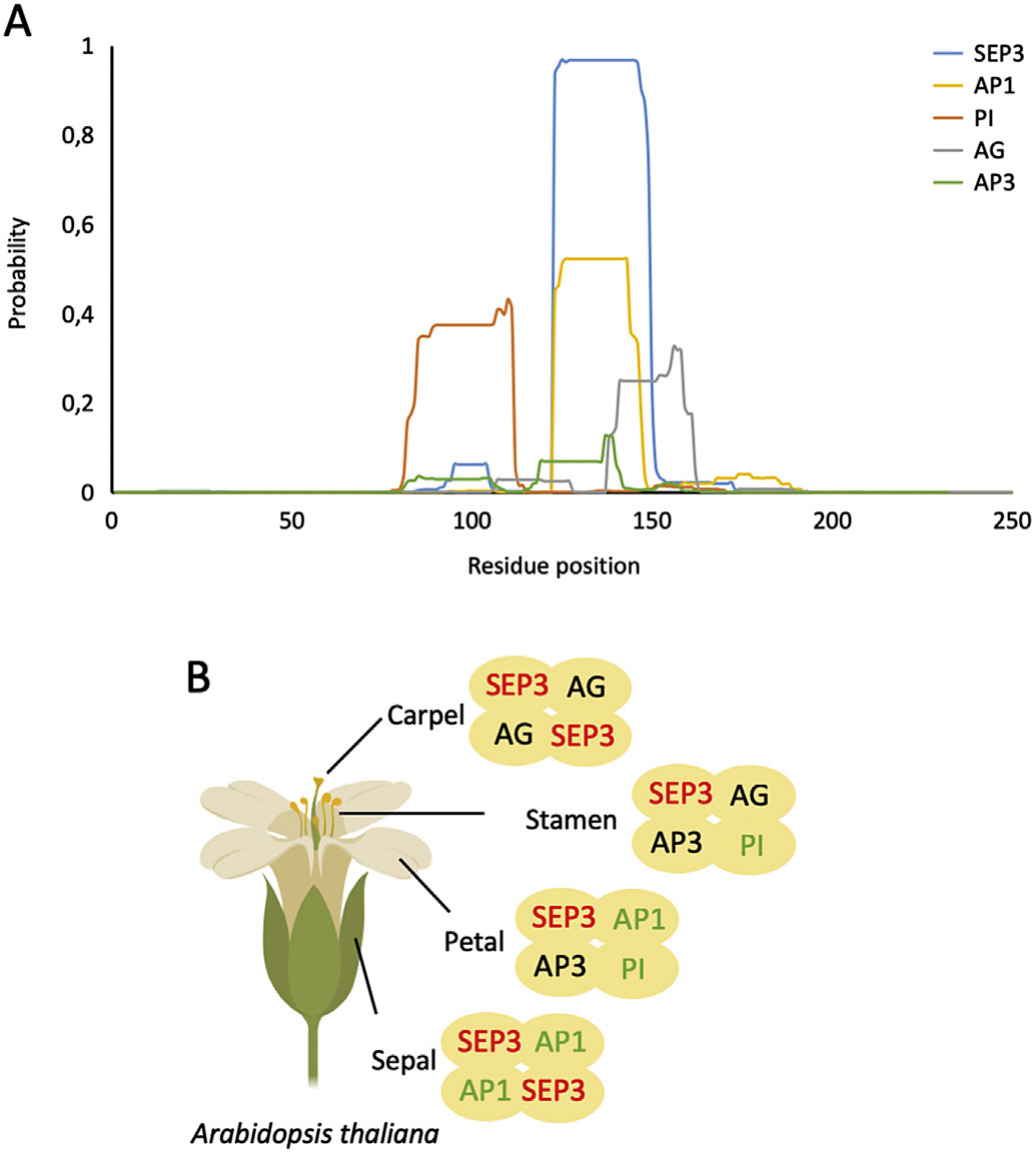
Coiled coil prediction of MADS TFs involved in flower organ specification in Arabidopsis. (A) The coiled coil probability was predicted by MultiCoil [66] using the full-length protein sequence of SEP3, AP1, PI, AG and AP3, respectively, as input. The prediction was set for coiled coil dimer formation with a prediction window of 21 residues. The peak regions correspond to the K domains of the corresponding sequences. (B) Schematic representation of flower quartet model in organ specification. SEP3 labels colored in red indicates strong probability of coiled coil formation, AP1 and PI in green medium, and AP3 and AG in black weak.

The K domain of SEP3 has a characteristic leucine zipper heptad repeat pattern, (abcdefg)_n_, in which the ‘a’ and ‘d’ positions are occupied preferentially by leucines but may comprise other hydrophobic residues (*e.g.* isoleucine and methionine). The leucine zipper is typical for coiled coils, which can be well predicted by bioinformatic algorithms, such as MultiCoil [66]. Using MultiCoil, we predicted the coiled coil formation probability of MADS TFs that are well studied in flower organ development in Arabidopsis, including SEP3, AG, AP3, PI and AP1 (Fig. 3A). These TFs are known to form the so-called floral “quartets”, in which specific heterotetrameric complexes formed by different MADS TFs specify the identity of the floral organs in each of the four whorls of a flower (Fig. 3B) [6,20]. Despite highly conserved primary sequences in the K domain (Fig. 2A), these MADS TFs show highly divergent probability to form coiled coils (due in large part to differences in key leucine residues in critical positions of the heptad repeats). SEP3, a well characterized MADS TF that acts as a hub for flower quartet formation [36], shows the highest probability to form coiled coils, consistent with its known stable coiled coil structure and promiscuity in forming complexes with other MADS TFs (Fig. 2A). This is followed by AP1 and PI (medium probability), and AG and AP3 (low probability) (Fig. 3A). Interestingly, PI coiled coil probability is high for the α1 helix involved in dimerization but low for the α2 helix important for tetramerization. Indeed, PI is not a hub MADS TF and seems to require additional partners such as SEP3 in order to form tetramers. At least one monomer in each potential quartet must have a strong coiled coil formation propensity in the α2 helix in order to drive tetramerization (Fig. 3B). Thus, the ability to form a stable coiled coil structural motif, at least in part, contributes to MADS TF complex formation. This modeling approach is potentially useful to analyze and explain why certain MADS TF complexes are able to form and are more stable/favored than others.

Based on the positions of hydrophobic residues in the heptad repeats of the K domain, recent studies have experimentally assessed the effects of these residues by means of mutagenesis coupled with size exclusion chromatography and electrophoretic mobility shift assays (EMSAs) [25,40,42]. Strikingly, even a single point mutation in SEP3 is sufficient to completely abrogate dimer/tetramer formation. For example, tetramerization was abrogated with alanine or valine mutations at positions L164 [42], L154 or L131, and dimerization abrogated with the leucine to arginine mutation, L115R [41]. In addition, full-length SEP3 with mutations to residues important for tetramerization was dramatically affected in its DNA binding cooperativity-instead of binding to two CArG boxes on a single DNA probe as a tetramer, the mutated proteins only bound to one site as a dimer. In contrast, increasing the stability of the tetramer through addition of leucine residues at the “a” position of the heptad repeat at positions 161 and 168 resulted in increased cooperativity [42]. Furthermore, a chimeric SEP3 construct consisting of part of the AP3 α2 helix mutated to preserve leucine residues at the “a” positions of the heptad repeat was able to homotetramerize and cooperatively bind DNA, unlike the AP3 wild type protein that is unable to homotetramerize [42]. The effect of tetramerization has also been tested *in planta* using a splice variant of SEP3 that lacks residues 161–174 in the α2 helix, which showed abrogated tetramerization activity *in vitro*. The transgenic plant expressing the tetramerization mutant of SEP3 in the *sep1sep2sep3* triple mutant background showed defects in flower meristem termination [50]. Taken together, these studies suggest that subtle changes in the MADS TF oligomerization domain affects their DNA-binding activity both *in vitro* and *in vivo* with accompanying changes in regulation of target genes.

#### 2.3.2 K Domain Tetramerization *Versus* Dimerization

A fundamental question remains as to what determines whether a MADS TF dimer will form an intramolecular coiled coil or recruit a second MADS TF dimer *via* the K domain to form a tetrameric complex. Based on extensive EMSAs studies and small-angle x-ray scattering experiments [41], it is becoming clear that only certain MADS TFs are able to act as drivers for tetramerization and that many MADS exist primarily as dimers. Based on sequence alignments, coiled coil predictions and homology modeling, certain MADS TFs likely act as tetramerization determinants due to specific residues between helices α1 and α2 which either favor or disfavor recruitment of a second MADS dimer for tetramer formation (Fig. 2B). SEP3 is able to form tetramers *in vitro* and *in vivo* and is one of the most promiscuous complex-forming MADS TFs [42]. Hub proteins, such as SEP3, act as central connecting nodes with many interaction partners in protein-protein interaction networks. Tetramerization capability may be a factor contributing to the characteristics of a hub protein for SEP3 and other MADS TFs [67]. Ancestral reconstructions of MADS genes reveals that SEP3 has retained this hub characteristic and even lost interactions over the course of evolution [68]. The SEP clade has highly conserved proline and glycine residues between α1 and α2 helices, which may favor an open conformation of the α1 and α2 helices and thus encourage tetramer formation with other MADS partners. SOC1, SVP, FUL and AP1 are additional examples of proteins that may be able to form tetramers in the MADS TF network and all have at least two glycine residues or a glycine and proline residue between the α1 and α2 helices [38,39,69]. The conservation of flexible amino acid motifs between the α1 and α2 helices may correlate with the propensity for inter *versus* intra molecular coiled coils to form (*i.e.* tetramerization), although this remains to be experimentally confirmed.

### 2.4 Carboxy-Terminal C Domain

The C-terminal domain of plant MIKC TFs has a variable primary sequence with few conserved structural motifs based on secondary structure prediction. However, some short amino acid motifs are present which likely play roles in target gene regulation and/or higher order complex formation. For example, the EAR motif (ethylene-responsive element binding factor-associated amphiphilic repression motif) with a consensus sequence LxLxL or DLNxxP is recognized by the *co*-repressors TOPLESS and SIN3-ASSOCIATED POLYPEPTIDE P 18 (SAP18). This motif is present in over 20 MADS TFs from Arabidopsis [70]. A direct interaction dependent on the EAR motif between the MADS TF, AGAMOUS-LIKE 15, and the corepressor, SAP18, has been demonstrated, suggesting that this interaction is important for the recruitment of a histone deacetylase complex [71]. Direct interactions with SEUSS, a co-repressor that forms a complex with LEUNIG, have been shown to require the C domain of SEP3 and AP1 [72]. Interestingly, transactivation activity has also been linked to the C-terminal domain for MADS TFs such as AP1 and SEP3 [[73], [74], [75]]. The presence of acidic, glutamine-rich and/or proline-rich clusters are often present in transcriptional activators and these types of clusters are present in both the AP1 and SEP3 C domains. These studies highlight the versatility of the C domain in mediating both activation and repression of target genes depending on the nature of the MADS TF complex and ternary factors.

For certain MADS TFs, the C domain does not appear to be required for function. For example, *AP3* or *PI* lacking the entire C domain were able to rescue *ap3* and *pi* mutant plants, respectively [76]. This further highlights the lack of conserved function of the C-terminal region. Higher order complex formation or stabilization of tetrameric complexes is another described role of the C domain [77]. Whether this is generally true in the MADS TF family requires further investigation.

## 3 Conclusions and Perspectives

The plant MADS TFs possess the highly conserved DBD that has exhibited little change over the course of evolution. A ubiquitous structural motif- the coiled coil- has been acquired by the MIKC plant MADS TFS that alters the oligomerization state and interaction patterns of the protein family, helping to explain the rich regulatory and functional diversity of these TFs. The K domain relies on leucine zipper-type interactions to alter complex formation in a myriad of ways *via* subtle changes in the energetics and kinetics of coiled coil formation. Initial formation of K domain α-helices for each monomer may depend on stabilizing hydrogen-bonding and salt bridge interactions, as has been experimentally shown for some coiled-coil forming proteins by NMR, time-resolved CD spectroscopy and mutagenesis [78]. These studies have not been performed to date for a K domain or K domain peptide from plant MADS TFs, but based on available structural data (SEP3 K domain, PDB 4OX0) residues on the hydrophilic face of the amphipathic K domain α-helices form a hydrogen-bonding network (R162/Q158/N155 and D152/Q148). In addition, the amino acids at these positions vary, suggesting a possible mechanism for controlling α-helix folding and stability. Additional amino acid substitutions on the hydrophobic face in each monomer α-helix in turn will stabilize or destabilize the formation of a dimeric coiled coil. Based on sequence alignments the “a” and “d” positions in MIKC MADS TFs vary, with leucine residues at the “d” position most strongly contributing to free energy stabilization.

Alternative splicing (AS) is another factor that increases plant MADS protein diversity by affecting primarily the oligomerization domains. The MIKC MADS proteins arise from multi-exonic genes, which undergo extensive AS resulting in alterations in the I and K domains that affects the PPI network and thus function. A number of predicted AS sites for the MADS family have been annotated, and are likely to be functionally relevant based on their location in the I/K domains and cross-species conservation [79]. Indeed, numerous transcripts corresponding to AS genes have been identified and physiological function of some of these isoforms has been determined. For example, in addition to the canonical transcript, SVP1, the SVP gene produces a shorter isoform, SVP3, that exhibits loss of interaction partners [52]. Overexpression of SVP1 and SVP3 in Arabidopsis result in different phenotypes, highlighting the function of AS in modifying PPI network and MADS activity [79]. Further examples include FLOWERING LOCUS M (FLM), in which different FLM isoforms are produced in a temperature dependent manner resulting in strong or highly attenuated DNA-binding when in complex with SVP [80]. Recent work from our team on SEP3 isoforms has also shown that AS changes the oligomerization capacity of the protein and results in changes in meristem determinacy and carpel development [50]. As more and more splice variants are being identified in RNA-seq datasets, it is becoming clear that the diversity of the MADS PPI network is even more complex than previously thought and changes in MADS function due to AS remains a rich subject to be explored.

Key questions remain as to MADS TF function and specificity. How do the different oligomerization patterns affect DNA binding and are there allosteric interactions which may further tune the direct interactions between the MADS DNA-binding domain and its cognate DNA? Is heterodimer or heterotetramer formation sufficient to functionally diversify the MADS TFs or are ternary factors necessary? How important is MADS tetramer cooperativity for gene regulation? Are additional oligomerization states accessible through extended coiled coil formation? Answers to these questions remain elusive, but the availability of structural, computational, modeling and genome wide binding data provide the necessary foundation for addressing these outstanding questions in the field.

## Acknowledgement

This work was supported by the Agence Nationale de la Recherche (project FloPiNet, ANR-16-CE92-0023) (CZ, VH, HD), Grenoble Alliance for Integrated Structural and Cell Biology (GRAL, financed within the University Grenoble Alpes graduate school (Ecoles Universitaires de Recherche) CBH-EUR-GS (ANR-17-EURE-0003)) (XL, VH, CZ) and CFR CEA thesis fellowship (AG).
